# Identification and functional characterization of the sulfakinin and sulfakinin receptor in the Chinese white pine beetle *Dendroctonus armandi*

**DOI:** 10.3389/fphys.2022.927890

**Published:** 2022-08-12

**Authors:** Bin Liu, Danyang Fu, Hang Ning, Ming Tang, Hui Chen

**Affiliations:** ^1^ College of Forestry, Northwest A&F University, Xianyang, China; ^2^ State Key Laboratory for Conservation and Utilization of Subtropical Agro-Bioresources, Guangdong Key Laboratory for Innovative Development and Utilization of Forest Plant Germplasm, College of Forestry and Landscape Architecture, South China Agricultural University, Guangzhou, China

**Keywords:** *Dendroctonus armandi*, sulfakinin, sulfakinin receptor, food intake, energy metabolism, RNA interference

## Abstract

The sulfakinin (SK) is an important signal molecule. As a neuromodulator, it mediates a variety of behavioral processes and physiological functions in invertebrates through the interaction with G-protein-coupled receptors (GPCRs). However, there is no report on the functional role of SK in the Chinese white pine beetle, *Dendroctonus armandi.* We have cloned and characterized *SK* and *SKR* genes in the *D. armandi* and carried out bioinformatics predictions on the basis of the deduced amino acid sequences, which are very similar to those from *Dendroctonus ponderosa.* The expression levels of the two genes were different between male and female adults, and there were significant changes in different developmental stages, tissues, and between starvation and following re-feeding states. Additionally, RNA-interference (RNAi) using double-stranded RNA to knock down *SK* and *SKR* reduced the transcription levels of the target genes and increased their body weight. In parallel, injection of SK caused a significant reduction in body weight and increase in mortality of *D. armandi* and also led to an increase in trehalose and a decrease in glycogen and free fatty acid. The results show that the SK signal pathway plays a positive and significant role in feeding regulation and provides a potential molecular target for the control of this pest.

## 1 Introduction

Sulfakinins (SKs) are multifunctional neuropeptides widely found in insects, which are similar to the mammalian peptides gastrin/cholecystokinin (CCK) in structure and function. The first insect sulfakinins (SKs) were isolated from the head extracts of the cockroach, *Leucophaea maderae* (Nachman et al., 1986a; Nachman et al., 1986b). They possessed a sulfated tyrosine residue in the characteristic C-terminal heptapeptide core sequence (DY(SO3) GHM/LRFamide) (Nachman et al., 1986a; Nachman et al., 1986b). Since then, SKs were widely identified in a variety of insect species, such as the fruit fly, *Drosophila melanogaster* (Nichols et al., 1988); American cockroach, *Periplaneta americana* (Veenstra, 1989); flesh fly, *Neobellieria bullata* (Fonagy et al., 1992); German cockroach, *Blattella germanica* (Maestro et al., 2001); giant mealworm beetle, *Zophobas atratus* (Marciniak et al., 2011); red flour beetle, *Tribolium castaneum* (Yu et al., 2013b); kissing bug, *Rhodnius prolixus* (Bloom et al., 2019); and brown planthopper, *Nilaparvata lugens* (Guo et al., 2021). Most studies on the function of insect SKs are carried out on the regulation of food intake (Wei et al., 2000; Downer et al., 2007; Yu et al., 2013b; Zels et al., 2015; Al-Alkawi et al., 2017; Guo et al., 2021), digestive processes (Nachman et al., 1997; Harshini et al., 2002; Zels et al., 2015; Guo et al., 2020), locomotion (Nichols et al., 2008; Chen et al., 2012), modulation of odor preferences (Nichols et al., 2008), aggression (Williams et al., 2014), synaptic growth (Chen and Ganetzky, 2012), sexual arousal (Wu et al., 2019), and energy metabolism (Slocinska et al., 2019; Slocinska et al., 2020).

However, the most significant effect described for SKs by far is their anorexic potency. Injection of SK notably reduced food intake in the desert locust, *Schistocerca gregaria* (Wei et al., 2000). In the blowfly, *Phormia regina*, SK injection caused a decrease in carbohydrate feeding (Downer et al., 2007). Additionally, injection of sulfated and nonsulfated analogs of SK also led to an inhibition of food intake in *T. castaneum* (Yu et al., 2013a; Yu et al., 2013b). The brown planthopper, *N. lugens*, injected with SKs consumed less food than the control (Guo et al., 2021). The satiety induction of SK was also proved by RNAi study in the Mediterranean field cricket, *Gryllus bimaculatus*, in which silencing of the SK resulted in an increase in food intake (Meyering-Vos and Mueller, 2007a). A large number of studies of RNAi in *D. melanogaster* showed that knock down of the SK in the brain led to an increase in food intake. The silencing of SK gene also affected the ability of *D. melanogaster* to distinguish different quality foods (Soderberg et al., 2012). Moreover, food uptake was induced by systemic silencing of the SK in *N. lugens* (Guo et al., 2021).

The SK signal system contains SK peptides and SK receptors (SKRs), which are similar to cholecystokinin receptors (CCKRs) in mammals, belonging to the G-protein-coupled receptor (GPCR). According to the sequence similarity to CCKRs, two SKRs named DSKR1 and DSKR2 have been cloned from *D. melanogaster* (Kubiak et al., 2002; Hauser et al., 2006). More studies have identified *SKR* genes in *Periplaneta americana* (Wicher et al., 2007), *T. castaneum* (Hauser et al., 2008), *R. prolixus* (Bloom et al., 2019), and yellow mealworm, *Tenebrio molitor* (Slocinska et al., 2020). In *T. castaneum*, the knock down of two *SKR* genes resulted in different degrees of changes in feeding behavior, which strongly proved that TcSKRs were involved in the control of food intake (Yu et al., 2013b; Yu and Smagghe, 2014b). In addition, the simultaneous silencing of the *SKR-1* and *SKR-2* in *R. prolixus* led to an increase in the mass of blood meal taken compared to controls (Bloom et al., 2019). Although some functional roles of CCK/SK signaling between insects and mammals seem to be conservative, the available data indicate that the underlying mechanisms are different (Nässel and Wu, 2022).

The Chinese white pine beetle, *Dendroctonus armandi* Tsai and Li (Coleoptera: Curculionidae: Scolytinae), is an endemic and destructive pest of coniferous forests in the middle Qinling Mountains of China, which not only attacks Chinese white pine, *Pinus armandii*, but also attracts other pests to the host plants, damaging the forest ecological system and causing heavy economic losses (Chen and Tang, 2007). Although SKs have been found in some insect species, there is no report on the functional roles of SK in *D. armandi.* In the present study, we cloned and identified full-length *SK* and *SKR* cDNAs from *D. armandi,* and also performed gene expression pattern analysis, related RNA interference, and peptide injection experiments. These results will serve as a vital step forward to improve the eco-friendly pest management strategies of bark beetles.

## 2 Materials and methods

### 2.1 Insect sample preparation

Chinese white pine beetles were obtained from infested *P. armandii* at the Huoditang Experimental Forest Station, which is located on the southern slopes of the mid-Qinling Mountains, Shaanxi, China (33°18′N, 108°21′E), and reared on an artificial food source in a laboratory maintained at 25 ± 1°C, 70% relative humidity (RH), and in the dark (Liu et al., 2021a).

### 2.2 cDNA cloning and sequencing

Total RNA was isolated from three developmental stages of the Chinese white pine beetle (larvae, pupae, and adults from both sexes), and the concentration and quality were determined as described previously (Liu et al., 2021a). Total RNA for cDNA synthesis was prepared using the Fast King RT Reagent Kit with gDNA Eraser (Tiangen, China) according to the manufacturer protocol. Partial sequences of *DaSK and DaSKR* were retrieved from the transcriptome database of *D. armandi* (Dai et al., 2015) and used for primer design (Supplementary Table S1). Full-length cDNA sequences of *DaSK and DaSKR* were obtained by 5′, 3′ RACE and confirmed as described previously (Liu et al., 2021a). Briefly, *DaSK* and *DaSKR* cDNA-specific primers for 5′ and 3′ RACE (Supplementary Table S1) were designed according to the obtained sequences, then the cDNAs were synthesized from RNA using a SMARTer RACE cDNA Amplification Kit (Clontech Laboratories Inc., Mountain, CA, United States) according to the manufacturer’s protocol. Touchdown PCR (annealing temperatures: 65–55°C) was used to amplify the 5′-UTR and 3′-UTR sequences. To obtain the full-length sequences, we designed specific primers containing the putative initiation and terminator codons (Supplementary Table S1).

### 2.3 Bioinformatic analysis

The two cDNA sequences obtained were deposited in the GenBank, and their accession numbers are listed in Table 1. The open reading frames (ORFs) of full-length cDNA sequences were obtained by ORF Finder (https://www.ncbi.nlm.nih.gov/orffinder/). DNAMAN 6.0 was used for multiple sequence alignment of proteins. Molecular weights (kDa) and isoelectric points were predicted using the ProtParam tool (Gasteiger et al., 2005). The putative signal peptide was predicted by Signal P 4.1 Server (https://www.cbs.dtu.dk/services/SignalP/). TMHMM v. 2.0 (https://www.cbs.dtu.dk/services/TMHMM/) was used to predict transmembrane domains. Phylogenetic trees were constructed using the software MEGA 6.0, employing the maximum-likelihood method with 500 bootstrap replicates (Tamura et al., 2013).

**TABLE 1:** Physicochemical properties of putative *D. armandi SK* and *SKR* proteins.

Gene name	Accession no.	ORF (bp)^a^	Amino acid residues^a^	MW (KDa)^a^	IP^a^
*SK*	MZ567222	354	117	13.69	6.82
*SKR*	MZ567223	1,248	415	47.47	9.31

a As predicted by the PROTPARAM program.

ORF, open reading frame; MW, molecular weight; IP, isoelectric point.

### 2.4 Insect sampling and treatments for RT-qPCR

*D. armandi* larvae were separated into two sub-stages: larvae (eating on host phloem for development) and mature larvae (ceased feeding). Pupae were also separated into two substages: early pupae (newly transformed from larvae) and late pupae (approach to becoming adults). Adults were separated into three substages: teneral adults, emerged adults, and feeding adults (Dai et al., 2014). The heads, foreguts, hindguts, midguts, thoraxes, fat body, and pheromone glands from emerged adults, and heads, foreguts, hindguts midguts, thoraxes, and body fat from larvae were isolated by dissection and then stored at −80°C until use.

The male and female emerged adults were divided into eight groups, and larvae were divided into seven groups. A group of collected insects were fed for 0 h as control and killed at time 0. The emerged adults of the other groups were immediately placed in glass dishes (2 cm high and 3 cm in diameter) with normal food in the artificial climate cabinet for 24 and 48 h. After feeding on a normal diet for 48 h, the adults and larvae were reared without food and starved for 72 and 48 h, respectively. Then, the alive beetles were subsequently re-fed for 24 h after starvation. Three samples were collected for each treatment.

The *CYP4G55* (accession number: KR012821.1) and *β-actin* (accession number: KJ507199.1) sequences of *D*. *armandi* were used as the reference genes for qRT-PCR (Vandesompele et al., 2002; Dai et al., 2014, 2015). Specific primers were used to detect the expression of *DaSK* and *DaSKR* (Supplementary Table S1). The qRT-PCR was performed as described in our previous study (Dai et al., 2014; Liu et al., 2021b). All assays were performed in three biological replicates, and the relative gene expression levels were calculated by the 2^−ΔΔCt^ method (Livak and Schmittgen, 2001).

### 2.5 RNA interference

#### 2.5.1 The dsRNA synthesis and injection

The T7 Ribo-MAXTM Express RNAi System (Promega, Madison, MI, United States) was used for the synthesis of dsRNA of *SK*, *SKR,* and green fluorescent protein (*GFP*) (GenBank accession: ACY56286). Primers (Table S1) were designed using Primer Premier 5.0 according to the obtained *SK* and *SKR* sequences. PCR amplification was performed, and the purified PCR products were used as a template for the synthesis of dsRNA. The final dsRNA products were diluted to 1,000 ng/µl with diethyl pyrocarbonate (DEPC)-treated water, then stored at –80°C. Afterward, each of the *D. armandi* emerged adults and larvae were microinjected with 0.15 µL dsRNA solution by Hamilton Microliter^TM^ syringes with 32 G needles (Hamilton, Bonaduz, Switzerland). Injection with dsGFP was used as a control. Each treatment group included 40 individuals, and 6 live beetles were collected at 24, 48, and 72 h after injection, then stored for qRT-PCR. Three samples were collected for each treatment.

#### 2.5.2 Body weight measurement

The body weight of *D. armandi* adults and larvae was recorded at different time points (24, 48, and 72 h) after injection. The beetles were left at room temperature after 1 h, and those no longer active were considered dead (Liu et al., 2021a). The weight of live beetles was determined by an electronic balance (d = 0.0001 g, Tianjin, AL204; Mettler-Toledo Ltd., China). Each measurement included three replicates.

### 2.6 Peptide injection

#### 2.6.1 Peptide synthesis and injection

Sulfakinin peptide was synthesized by Sangong Biotech (Shanghai, China). Peptide weight was determined by MALDI-TOF mass spectrometry and amino acid analysis was used to quantify the amount of peptide. The amino acid sequence of the peptide used in this study was as follows: sulfated sulfakinin peptide (sSK): EEQVDDY(SO_3_H) GHMRFamide. Before injection, the peptide was dissolved in phosphate buffer saline (PBS). Each of the *D. armandi* emerged adults and larvae were microinjected with 0.2 µl of peptide solution (2.0 pmol/insect). Beetles injected with the same volume of PBS were used as control. Then, they were kept in an artificial climate cabinet under normal condition. Each treatment group contained 40 individuals, and three samples were collected for each treatment.

### 2.7 Survival test and body weight determination

*D. armandi* adults and larvae mortality was recorded at different time points (12, 24, 36, 48, 60, and 72 h) after injection. Then, left at room temperature for 1 h; the beetles that were no longer active were considered dead. The body weight of the live beetle was determined as previously described.

### 2.8 Measurement of glycogen, free fatty acid, and trehalose

For *D. armandi* emerged adults and larvae at 24, 48, and 72 h after injection, we determined three physiological indices of beetles, namely the contents of glycogen, trehalose, and free fatty acid, according to appropriate methods as previously described (Liu et al., 2021a). Briefly, whole-body homogenates of each group were used to measure glycogen, trehalose, and free fatty acid. The three content levels were measured with a spectrophotometer (UV-1800PC, Shanghai Mapada Instrument Co., Ltd., Shanghai, China) using the relevant kits (TY-2-Y for glycogen, FFA-1-W for free fatty acid, and HT-2-Y for trehalose;, SuzhouComin Biotechnology Co., Ltd., Jiangsu, China). Three biological replicates (six beetles for one replicate) were performed for each sample.

### 2.9 Statistical analysis

All statistical data analyses were performed using SPSS Statistics 19.0 (IBM, Chicago, United States). Student’s t-test was used for pairwise comparisons, and one-way ANOVA was used for comparisons among multiple groups, followed by Tukey’s multiple comparisons. Graphs were plotted using Prism 6.0 (GraphPad Software, CA, United States).

## 3 Results

### 3.1 Sequencing and bioinformatics analysis

The full-length *D. armandi SK* and *SKR* cDNAs were cloned and analyzed. The lengths of the coding regions of *SK* and *SKR* are 354 bp and 1,248 bp, which encode 117 and 415 amino acid residues, respectively. In addition, the molecular weights (MW) of the deduced proteins of SK and SKR are 13.69 and 49.47 kDa, and the isoelectric points (PI) are 6.82 and 9.31, respectively (Table 1).

**FIGURE 1:**
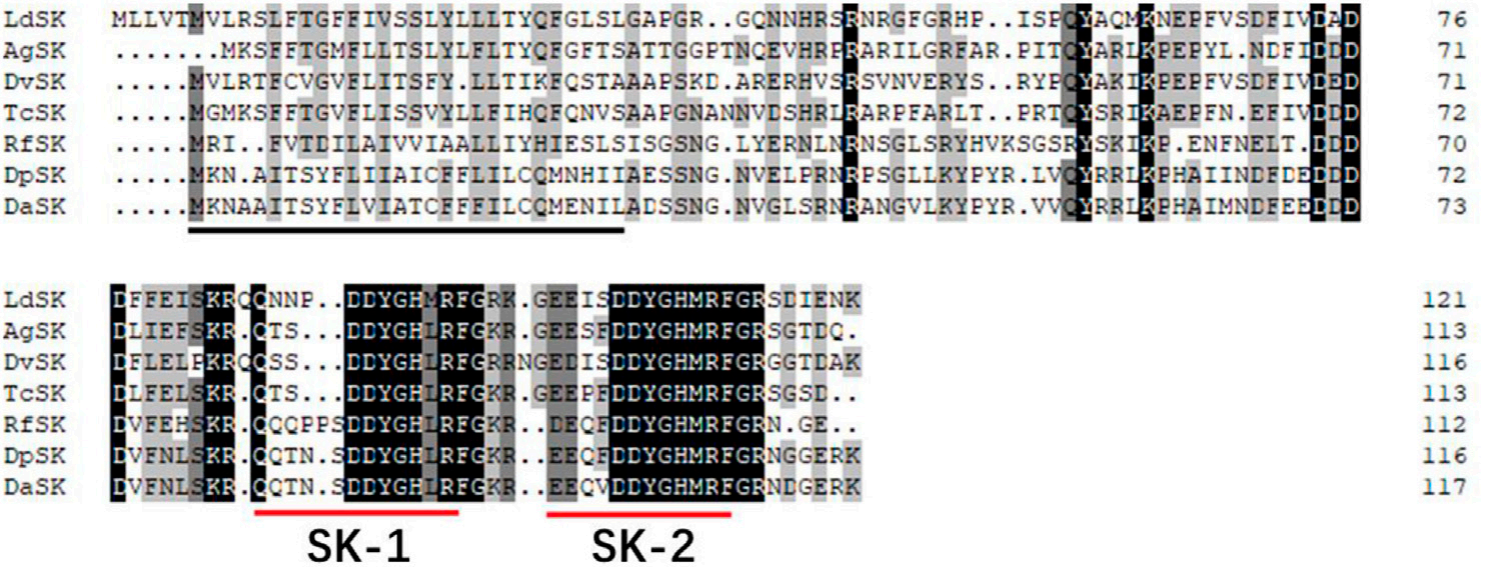
Deduced amino acid sequence of *D. armandi* SK and comparison of the amino acid sequence of the SKs with those of other species. They include *Dendroctonus ponderosae* (DpSK), *Rhynchophorus ferrugineus* (RfSK), *Tribolium castaneum* (TcSK), *Diabrotica virgifera virgifera* (DvSK), *Anoplophora glabripennis* (AgSK), and *Leptinotarsa decemlineata* (LdSK). The putative signal region is indicated by a solid black line, and the regions corresponding to two SKs are underlined by a solid red line. Identical amino acid residues in all proteins are shown in black, and grey parts indicate similar amino acids.

**FIGURE 2:**
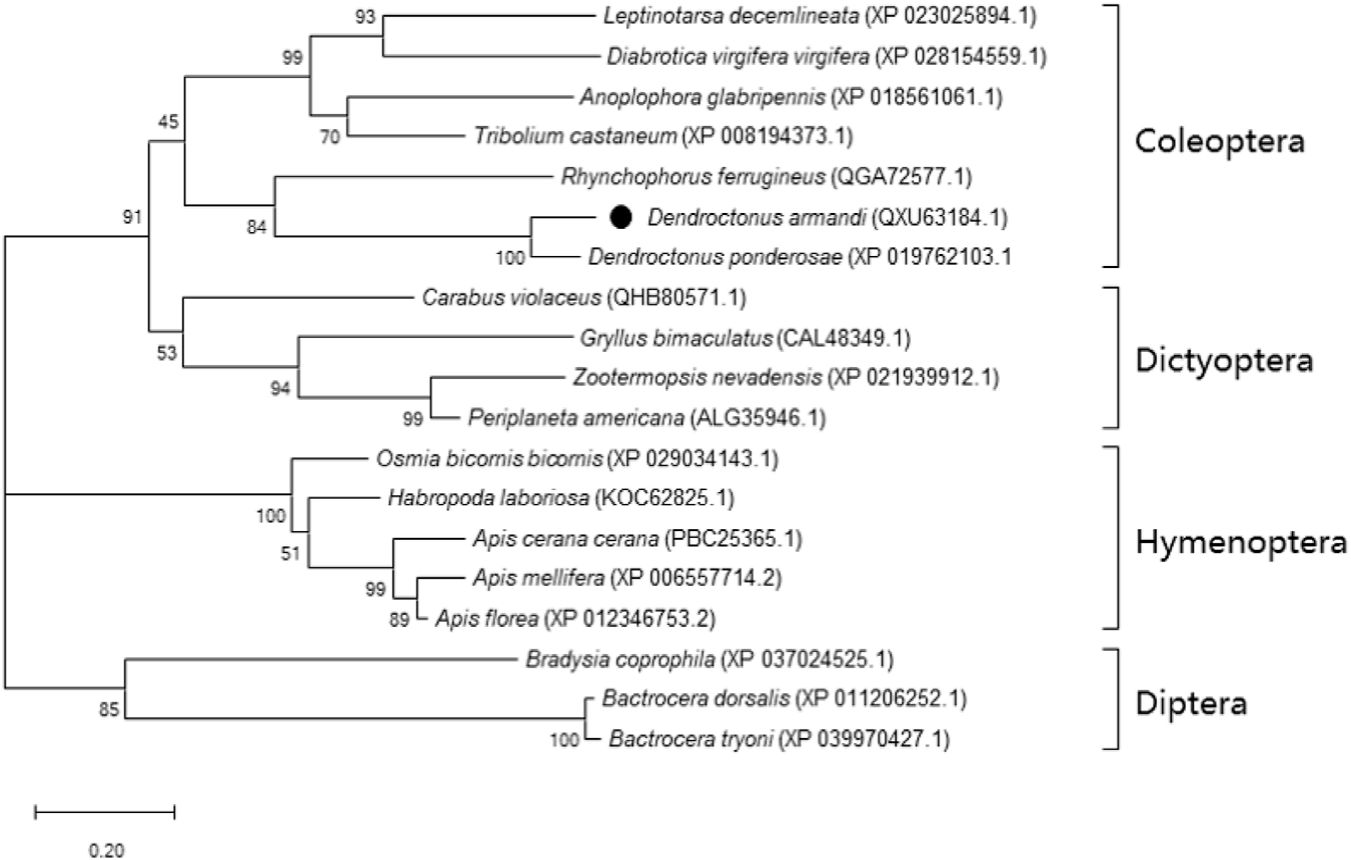
Phylogenetic analysis of DaSK with other insect species. The phylogenetic tree was constructed by the maximum-likelihood method using the amino acidic substitution model WAG in MEGA 6.0. Bootstrap values (500 replicates) are indicated next to the branches and GenBank accession numbers are shown in parentheses. The black dot indicates *D. armandi* SK.

The SK precursor of *D. armandi* contained 117 amino acids, including the first 28 amino acids, which were predicted to be a signal peptide, and the other two peptides that were designated DaSK-1 and DaSK-2. Both peptides contained characteristic Tyr and Gly that are potential sulfation and amidation sites, respectively (Figure 1). The protein sequence of SKR showed the typical characteristic of the rhodopsin-like GPCR family including seven transmembrane domains (Supplementary Figure S1). DaSK and DaSKR had the highest similarity with the same proteins from *Dendroctonus ponderosa* (Supplementary Table S2). The phylogenetic trees of SK (Figure 2) and SKR (Supplementary Figure S2) showed that they were clustered with the Coleoptera group.

### 3.2 RT-qPCR

#### 3.2.1 Expression of genes in different developmental stages and tissues

**FIGURE 3:**
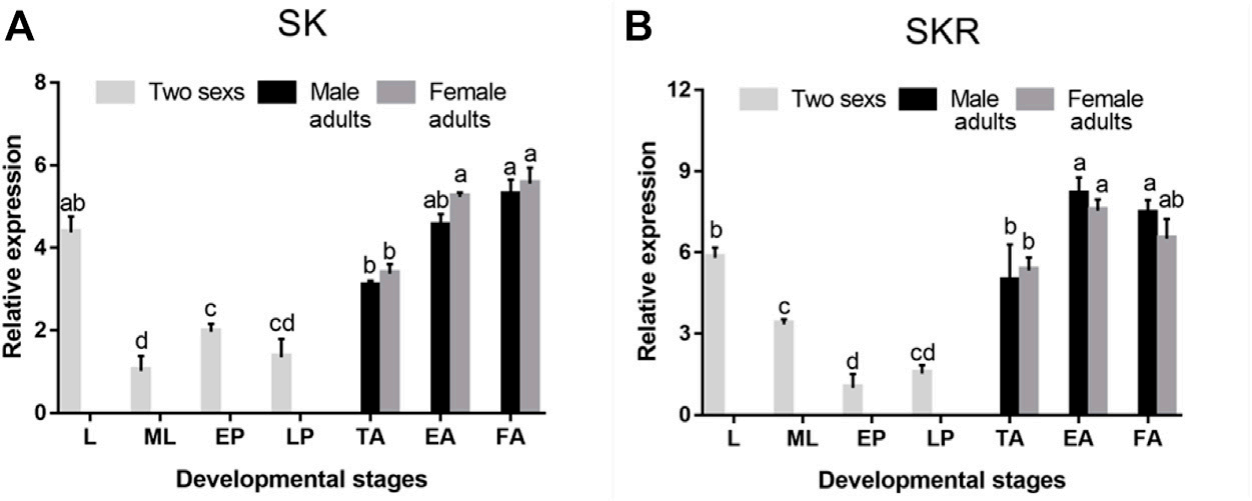
Relative mRNA expression levels of *SK* **(A)** and *SKR* **(B)** in different developmental stages of *D. armandi*. The relative expression levels were normalized with *β-actin* and *CYP4G55*. Different lowercase letters indicate significant differences at the 0.05 level. (one-way ANOVA, Turkey test). All values are mean ± *SE*, *n* = 3. L, larvae; ML, mature larvae; EP, early-stage pupae; LP, late-stage pupae; TA, teneral adults; EA, emerged adults; FA, feeding adults.

*SK* and *SKR* were expressed in all developmental stages of *D. armandi*, but showed different patterns. They were expressed the highest in the adult stage, followed by an expression in the larval stage, while *SK* and *SKR* expressions were the lowest in mature larvae and early pupae, respectively (Figures 3A,B). In addition, no statistically significant differences were found between male and female adults.

**FIGURE 4:**
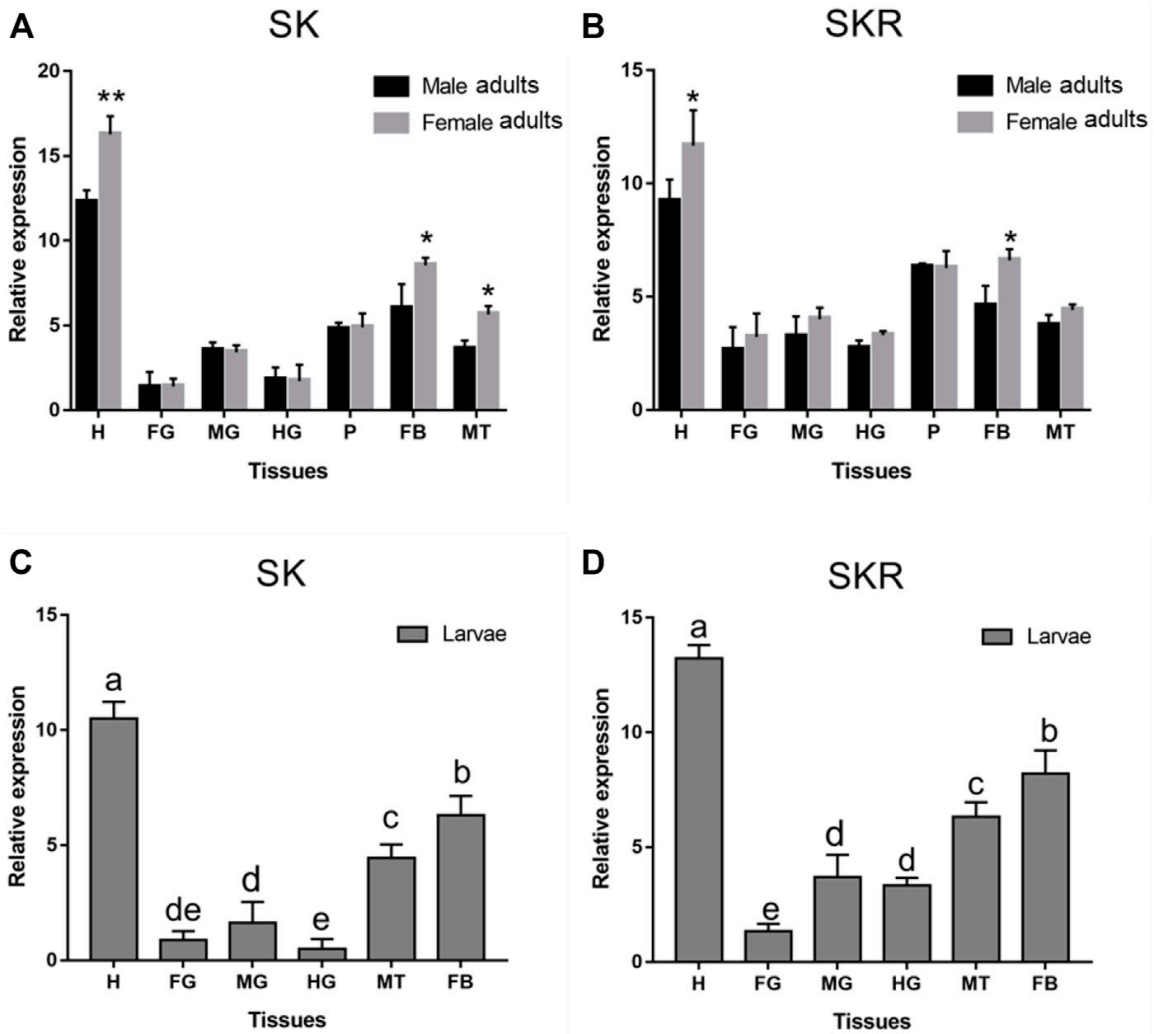
Relative expression levels of *SK* **(A)** and *SKR* **(B)** in emerged adults and *SK* **(C)** and *SKR* **(D)** in larvae in the different tissues of *D. armandi*. The relative expression levels were normalized with *β-actin* and *CYP4G55*. Different lowercase letters indicate significant differences at the 0.05 level. The asterisk indicates a significant difference between female and male expression levels (*p < 0.05 and **p < 0.01, independent Student’s t-test). All values are mean ± *SE*, *n* = 3. H, head; FG, foregut; MG, midgut; HG, hindgut; P, pheromone gland; MT, Malpighian tubules; FB, fat body.

*SK* and *SKR* of *D. armandi* were expressed in different tissues at different levels, and there were sex differences in some tissues (Figure 4). They had the highest expression in the head and the lowest expression in the foregut. Moreover, both of them were also highly expressed in the Malpighian tubules and fat body. *SK* had a higher expression in females than males in the head, Malpighian tubules, and fat body (Figures 4A,B), while *SKR* was highly expressed in the head and fat body, with a significantly higher expression in females than in males in the two tissues (Figures 4C,D).

#### 3.2.2 Starvation and re-feeding assays

**FIGURE 5:**
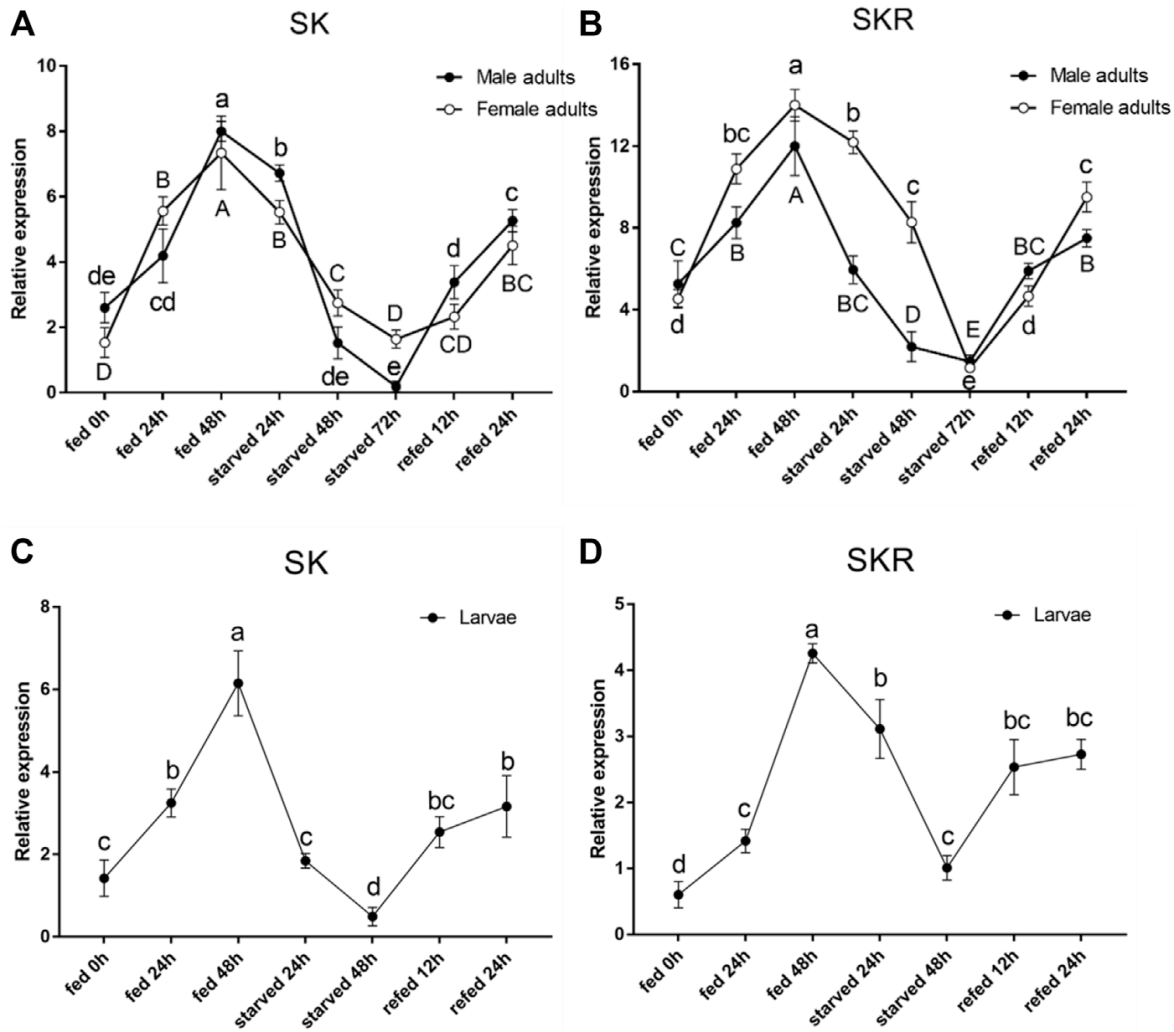
Relative expression levels of *SK* **(A)** and *SKR* **(B)** in emerged adults and *SK* **(C)** and *SKR* **(D)** in larvae after starvation and subsequent re-feeding treatment in *D. armandi*. The relative expression levels were normalized with *β-actin* and *CYP4G55* using the expression levels in the 0 h for calibration. Different letters indicate significant differences at the 0.05 level (uppercase for males, lowercase for females, uppercase for males and larvae, and no letter means no significant difference among all time points). All values are mean ± *SE*, *n* = 3.

The expression levels of both *SK* and *SKR* indicated a similar response to starvation and re-feeding assays. The *SK* and *SKR* expression levels in *D. armandi* adults (Figures 5A,B) and larvae (Figures 5C,D) were notably down-regulated in the starved groups compared with the feeding groups and reached the lowest at 72 h. Moreover, during the re-fed experiment after food deprivation, the *SK* and *SKR* expression levels showed a steady increase.

### 3.3 Efficiency analysis of RNAi

#### 3.3.1 Effect of dsRNA injection on *SK* and *SKR* expression level

**FIGURE 6:**
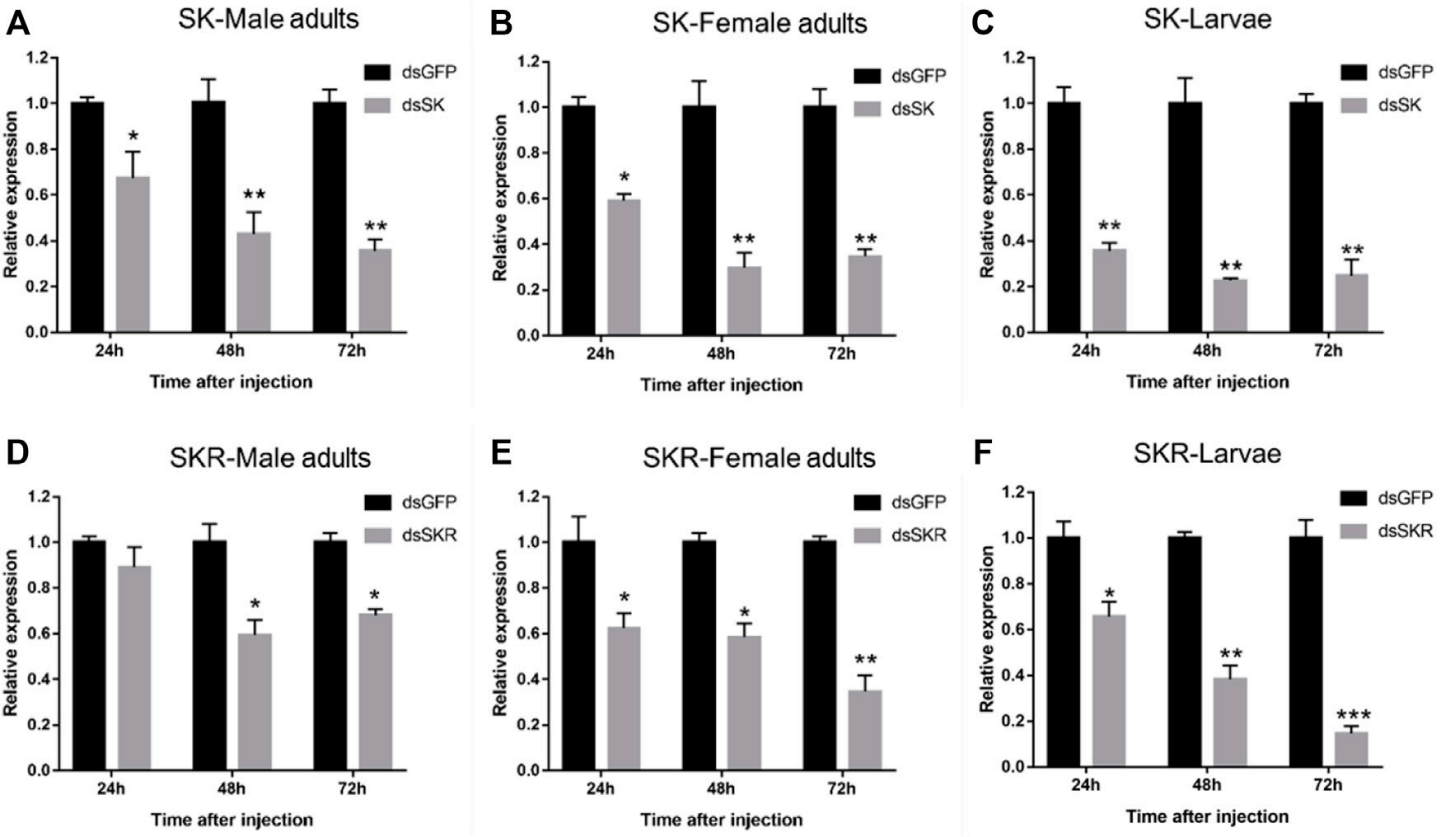
Relative expression levels of SK-Male adults **(A)**, SK-Female adults **(B)**, SK-Larvae **(C)**, SKR-Male adults **(D)**, SKR-Female adults **(E),** and SKR-Larvae **(F)** in *D. armandi* at 24, 48, and 72 h after dsRNA injection. The relative expression levels were normalized with *β-actin* and *CYP4G55*. The asterisk indicates a significant difference between dsRNA treatment group and the control group (*p < 0.05, **p < 0.01, and ***p < 0.001, one-way ANOVA). All values are mean ± *SE*, *n* = 3.

Compared with the control group, the expression levels of *SK* (Figures 6A–C) and *SKR* (Figures 6D–F) in *D. armandi* adults and larvae were notably down-regulated at 24, 48, and 72 h after dsRNA injection, except for the expression level of *SKR* in male adults at 24 h (Figure 6D). Moreover, the expression level of *SK* decreased most in male adults, female adults, and larvae, which reached 64.3, 70.3, and 77.3% (the 100% reference), respectively. While that of *SKR* was 40.7, 65.3, and 85.3%, respectively.

#### 3.3.2 Effect of dsRNA injection on body weight

**FIGURE 7:**
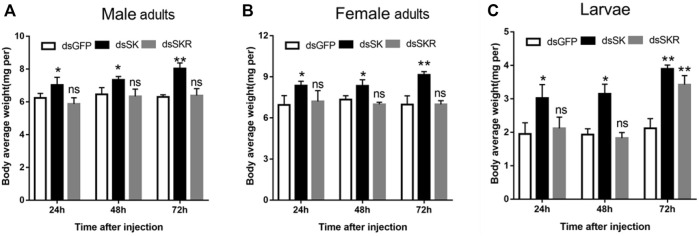
Effect of dsSK and dsSKR on body average weight of male adults **(A)**, female adults **(B)**, and larvae **(C)**. Samples were collected and assayed at 24, 48, and 72 h after injection. The asterisk indicates a significant difference between treatments (*p < 0.05 and **p < 0.01, ns, not significant, one-way ANOVA). All values are mean ± *SE*, *n* = 3.

Compared with the control group, the average weight of *D. armandi* adults and larvae significantly increased at 24, 48, and 72 h after injection of dsSK (Figure 7); among them, the male adults and larvae had the least and most weight gain, with 21.5 and 45.7%, respectively (Figures 7A,C). Particularly, the average body weight of beetles injected with dsSKR did not change obviously, except for the larvae at 72 h (Figure 7).

### 3.4 Efficiency analysis of SK peptide

#### 3.4.1 Effect of SK peptide injection on mortality and body weight

The mortality of *D. armandi* larvae and adults after SK peptide injection was higher than the control group (Supplementary Figure S3). From 0 to 72 h, after larvae and adults were injected with SK peptide, their mortality significantly increased. The highest mortality was observed at 72 h, Moreover, the mortality of female adults was the lowest and larvae was the highest, with 66.7 and 83.3%, respectively (Supplementary Figure S3A,C).

**FIGURE 8:**
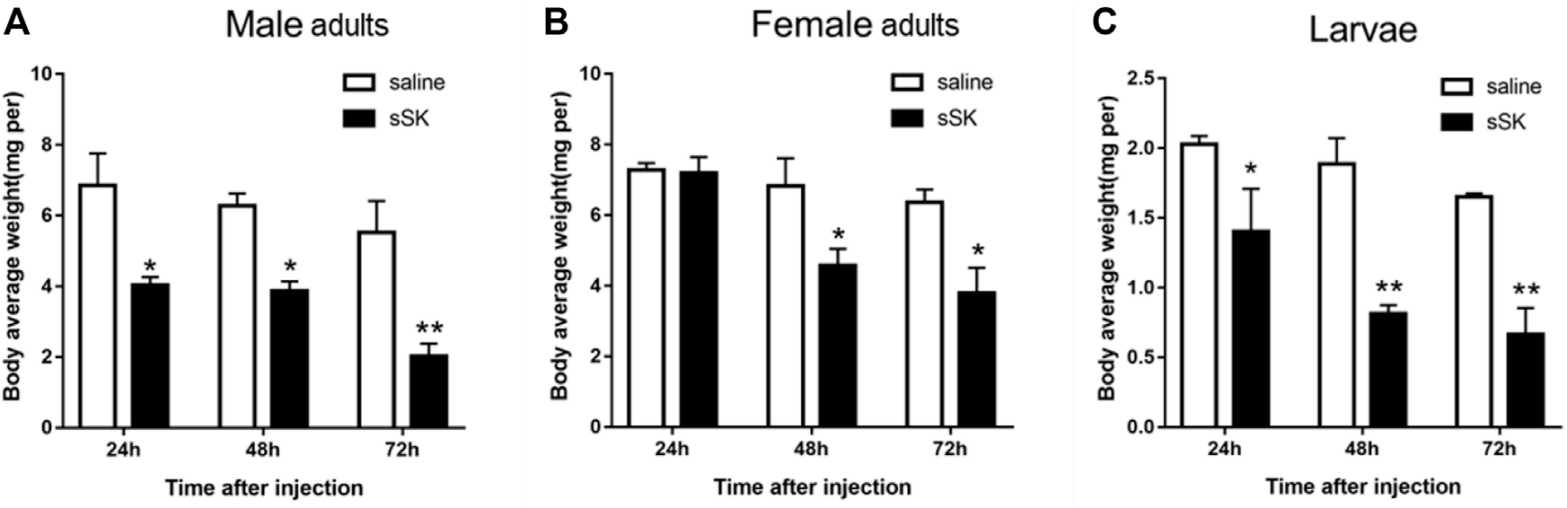
Effect of sSK peptide on body average weight of male adults **(A)**, female adults **(B)**, and larvae **(C)**. Samples were collected and assayed at 24, 48, and 72 h after injection. The asterisk indicates a significant difference between treatments (*p < 0.05 and **p < 0.01, one-way ANOVA). All values are mean ± *SE*, *n* = 3.

Compared with the control group, the average weight of *D. armandi* larvae and adults significantly decreased at 24, 48, and 72 h after injection of SK peptide (Figure 8), except for the female adults at 24 h (Figure 8B). Among them, the female adults and the larvae had the least and the most weight loss, with 33.2 and 57.9%, respectively (Figures 8B,C).

#### 3.4.2 Effects of SK peptide injection on regulating energy metabolism

**FIGURE 9:**
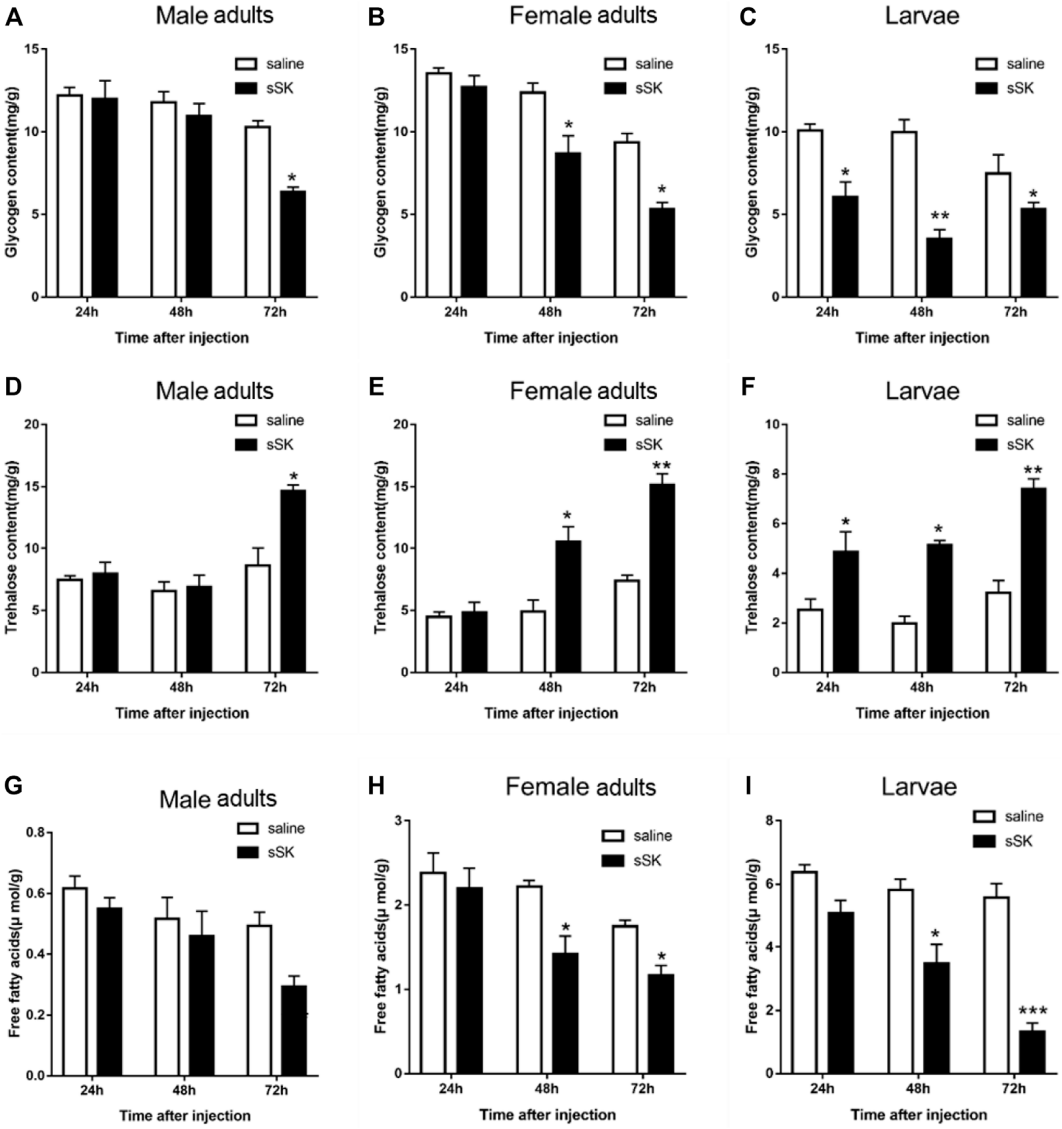
Effect of SK on energy metabolism. Whole body was used to determine the contents of glycogen in male adults **(A)**, female adults **(B),** and larvae **(C)**; free fatty acids in male adults **(D)**, female adults **(E),** and larvae **(F);** and trehalose in male adults **(G)**, female adults **(H),** and larvae **(I)**. Samples were collected at 24, 48, and 72 h after injection. The asterisk indicates a significant difference between treatments (*p < 0.05 and **p < 0.01, and ***p < 0.001, one-way ANOVA). All values are mean ± *SE*, *n* = 3.

The contents of glycogen and free fatty acid in *D. armandi* adults were significantly decreased after injection of SK peptide at 48 and 72 h compared with the control group (Figure 9), except for the male adults at 48 h (Figures 9A,G), while in larvae were significantly decreased at 24, 48, and 72 h, except for free fatty acid content at 24 h (Figure 9I). Among them, the largest increase was observed at 72 h after injection. It is worth noting that the trehalose content of male and female adults and larvae increased most at 72 h after injection of SK peptide compared with the control group, reaching 41.1%, 51.0, and 56.6%, respectively (Figure 9).

## 4 Discussion

In this study, full-length coding sequences of genes *SK* and *SKR* were amplified from *D. armandi* and the physicochemical properties of translated proteins were analyzed. When the deduced DaSK precursor was compared with the amino acid sequences of other insect SK proteins, it was found that the regions corresponding to these two SK peptides were relative highly conserved together with the dibasic cleavage sites and amidation signal at C-terminal, while the rest were less conserved. This is also consistent with the results obtained in previous research (Meyering-Vos and Mueller, 2007b; Duve et al., 2010; Yu et al., 2013b). Moreover, DaSKR showed high similarity to those of other predicted SKRs, which had some conserved characteristic residues in the seven transmembrane domains, an extracellular N-terminal region, and a cytosolic C-terminus (Costanzi, 2012).

We observed that both the *SK* and *SKR* were expressed throughout various stages of *D. armandi*, indicating that the SK signaling system is may be involved in regulating some physiological processes in the development and growth of beetles. A similar result was observed in *T. castaneum*, in which the *SK* and *SKR* were highly expressed in the head of both larvae and adults, and this phenomenon was most obvious in the larval stage (Yu et al., 2013b; Yu and Smagghe, 2014b). Previous studies have shown different expression levels of *SK*, *SKR1,* and *SKR2* in the head, gut, and other remaining tissues of *T. castaneum* larvae and adults (Yu et al., 2013b; Yu and Smagghe, 2014b). *SK* transcript of *R. prolixus* was also detected in neurons of the brain of males and females, as well as in the cricket *G. bimaculatus* ((Meyering-Vos and Mueller, 2007a; Bloom et al., 2019). In addition, *SKR*s in *T. molitor* were identified not only in the ventral nerve cord and brain but also in peripheral tissues such as the gut, fat body, and hemolymph (Slocinska et al., 2019; Slocinska et al., 2020). This was in line with the situation in *D. armandi.* In addition to the head, *DaSK* and *DaSKR* expression was observed in several other tissues, such as the Malpighian tubules, fat body, pheromone gland, and gut tissues. Obviously, *SK* was highly expressed in the head, indicating its role as a neurotransmitter. Therefore, we speculate that SK could be transmitted to other tissues where it might play a significant role in various physiological processes.

In the present study, we observed that with a prolonged starvation time, the transcription level of *SK* in *D. armandi* decreased, but the subsequent re-fed led to a continuous increase. The starvation and re-feeding experiments provided direct evidence for the effect of SK signaling system on feeding behavior. This result was similar to that found in *N. lugens.* The expression level of *SK* significantly increased when they were re-fed after starvation (Guo et al., 2021). The expression pattern of *SKR* in the starvation and re-fed experiments was in line with *SK*. These results indicate that the state of feeding has an important influence on the expression levels of the *SK* and *SKR* in *D. armandi*.

To further explore the function of the SK signal pathway in the beetle feeding, we knocked *SK* and *SKR* down in larvae and adults through RNAi technology. The results showed that it could effectively inhibit the expression of *SK* and *SKR*, while the duration and efficiency of the silencing effects were distinct. Silencing of *SK, SKR1,* and *SKR2* by RNAi has been related to the stimulation of food uptake in *T. castaneum and R. prolixus* (Yu et al., 2013b; Yu and Smagghe, 2014b; Al-Alkawi et al., 2017)*.* In contrast to the increase in food intake in the RNAi assays, injection of sSK peptide may have strengthened the SK signal and significantly suppressed food intake in the larvae of *T. castaneum* (Yu et al., 2013b). Similarly, injection of SK-1 resulted in taking smaller blood meals in *R. prolixus* (Al-Alkawi et al., 2017)*,* which is in accordance with the present study in *D. armandi*. The beetles had a lighter body weight after SK peptide injection, while the opposite results were observed after RNAi of *SK* and *SKR*. Additionally, the mortality was notably higher in larvae and adults than in control after SK peptide injection. We speculate that SK might lead beetles to eat less, causing a delay in growth and development, resulting in a decrease in body weight and ultimately to the death of the *D. armandi*. This indicated that SK injection suppressed *D. armandi* appetite, likely resulting in a change in food intake, which shows that SK may be a critical factor to regulate feeding.

A previous study has reported that the SK signaling system was regulated by the components of the insulin signal pathway in *D. melanogaster* (Soderberg et al., 2012). In the present study, we found that the *D. armandi* SK signaling regulated feeding by affecting energy metabolism, in which injection with SK led to an increase in trehalose and a decrease in glycogen. Presumably, with less food or even starvation, SK signaling system not only promotes energy storage or biosynthesis but also inhibits energy utilization or metabolism. This pattern was consistent with the observation from *Z. atratus* (Slocinska et al., 2015). However, the opposite effect was observed in the larvae of *T. molitor;* decreasing tendency was found in trehalose content after SK application, while glucose level increased (Slocinska et al., 2020). This maybe shows the species-specific action of SK in different insects. Moreover, the level of insulin-like peptides in the larvae of *T. molitor* increased after SKs administration (Slocinska et al., 2020), indicating that the SK signaling system was involved in insulin signals to regulate the process of energy metabolism, which is a hypothesis to be further studied in *D. armandi*.

Interestingly, only one *SKR* was found in *D. armandi* (Dai et al., 2015), while two *SKR*s had been functionally characterized in other insect species (Hauser et al., 2008; Bloom et al., 2019; Slocinska et al., 2020). Whether there is another receptor in *D. armandi* needs further study. In particular, SK signaling system was missing in the pea aphid, *Acyrthosiphon pisum* (Yu and Smagghe, 2014a)*.* Previous studies speculated that the lack of SK signaling may promote food intake and increase the energy supply of insects as *A. pisum* secrete honeydew, resulting in a large loss of energy (Li et al., 2013). Because the interaction of SK and SKR initiates the SK signaling, Yu et al. (2015) demonstrated that SKR1 was more flexible than SKR2 during the ligand–receptor interactions in *T. castaneum*.

In conclusion, the cDNAs of *DaSK* and *DaSKR* have been cloned and functionally identified. The wide temporal and spatial distribution of the *DaSK* and *DaSKR* expression highlights their importance in regulating feeding processes and other potential roles in reproduction and energy metabolism. Additionally, silencing of *SK* and *SKR* reduced the transcription levels of the target genes and increased their body weight. In parallel, injection of SK led to a significant reduction in body weight and an increase in mortality of *D. armandi*. The information on regulating body weight by the SK signal pathway provides a potential new target for developing new pest control strategies. However, it is not clear how SK/SKR signal system modifies feeding-related processes at the molecular, neural circuit, and cellular levels in *D. armandi.* Further studies are needed to more clearly explain the mechanisms involved in feeding regulation.

## Data Availability

The datasets presented in this study can be found in online repositories. The names of the repository/repositories and accession number(s) can be found in the article/Supplementary Material.
