# Supplementary material for: Identification and functional characterization of the sulfakinin and sulfakinin receptor in the Chinese white pine beetle Dendroctonus armandi
Source: Front Physiol. 2022 Aug 12;13:927890. doi: 10.3389/fphys.2022.927890 (PMC9417412; doi:10.3389/fphys.2022.927890)
Supplement: Supplementary file 1 [file DataSheet1.docx]

Supplementary Material

**Table S1** Primer sequences used in the research

| Gene | Sequence （5′ → 3′） | | purpose |
| --- | --- | --- | --- |
|  | Forward | Reverse |  |
| *SK* | CAATACAGAAGATTAAAACCT | CCTACCGAACCTCATATG | cDNA |
|  | GAAAACATTCTTGCGGACTC | GAAGAAGACGATGACGATGT | 3’RACE |
|  | AATGGTAATGTTGGACTTTCA | AAGACGATGACGATGTATTTA | 5’RACE |
|  | AACGCCATTACAAGCTACTT | CACCACCATTCCTACCGAAC | Full-length |
|  | GCTACTTCCTAGTTATTGCCAC | ATCGTCTTCTTCAAAATCGTTC | qPCR |
|  | TAATACGACTCACTATAGGGGCTACTTCCTAGTTATTGCCAC | TAATACGACTCACTATAGGGCGTCTTCTTCAAAATCGTTCAT | RNAi |
| *SKR* | CGTTTCCGATCTGCTTCTTG | TGAAGCAGTAGGTGATGGGA | cDNA |
|  | AGAGGCTGGTGAGCGGGTTG | CGTCCTCTTCACCGACCAAT | 3’RACE |
|  | CGCTTACCGCCACTAAACAA | TTGCCTTTGATGGTGGGTTT | 5’RACE |
|  | GGAGGAAATTCAGCATGTTCGACAT | TCTCTAGATCAGCGCCGGTTAATTT | Full-length |
|  | CGGCTCCAACAGCTCCAAGAG | CAGCACCACCACGCACAACAT | qPCR |
|  | TAATACGACTCACTATAGGGGAAACCCACCATCAAAGGCA | TAATACGACTCACTATAGGGAGAACTCCAGCACCACCACG | RNAi |
| *GFP* |  |  |  |
|  | TAATACGACTCACTATAGGGATGGTGTTCAATGCTTTTCA | TAATACGACTCACTATAGGGCTCTCTTTTCGTTGGGGTCT | RNAi |

**Table S2** Amino acidic identity of SK and SKR cDNAs isolated from *D. armandi* with related sequences in other insect species

| **Gene** | **BLAST matches in GenBank** | | |  |
| --- | --- | --- | --- | --- |
| **Name** | **Species** | **Name** | **Accession Number** | **Identity^a^ %** |
| *SK* | *Dendroctonus ponderosae* | drosulfakinins | XP_019762103.1 | 85 |
|  | *Tribolium castaneum* | drosulfakinins | XP_008194373.1 | 49 |
|  | *Rhynchophorus ferrugineus* | drosulfakinins | QGA72577.1 | 41 |
| *SKR* | *Dendroctonus ponderosae* | cholecystokinin receptor-like | XP_019756917.1 | 90 |
|  | *Rhynchophorus ferrugineus* | cholecystokinin receptor-like | QGA72519.1 | 68 |
|  | *Sitophilus oryzae* | cholecystokinin receptor-like | XP_030750622.1 | 66 |

Note: ^a^ As predicted by BLAST (http://www.ncbi.nlm.nih.gov).


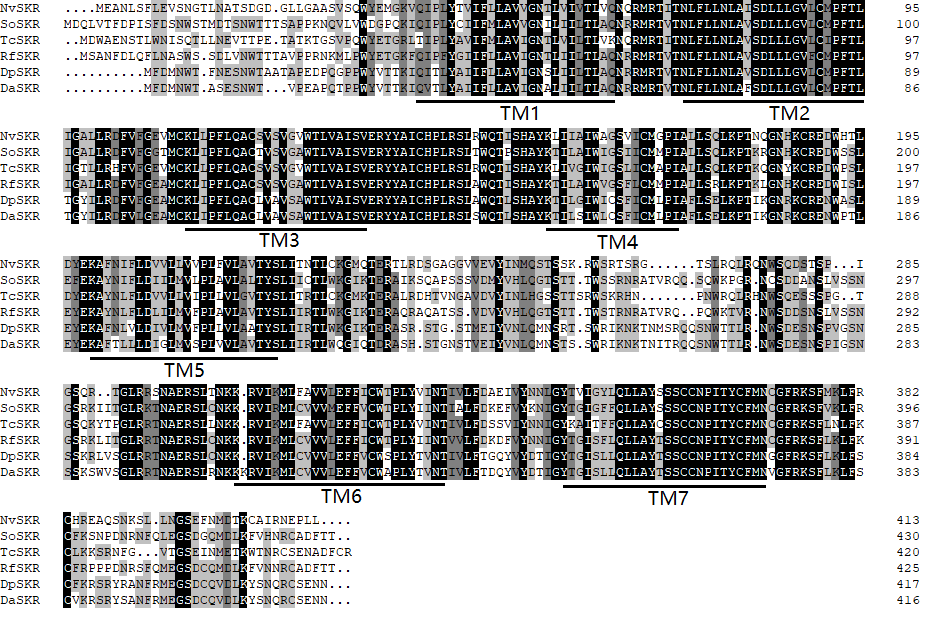


**Figure S1** Comparison of the amino acid sequence of *D. armandi* SKR with those of other species. They include *Dendroctonus ponderosae* (DpSKR), *Rhynchophorus ferrugineus* (RfSKR), *Tribolium castaneum* (TcSKR), *Sitophilus oryzae* (SoNPFR) (SoSKR) and *Nicrophorus vespilloides* (NvSKR) The predicted seven transmembrane domains are underlined by a solid line. Identical amino acid residues in all proteins are shown in black, grey parts indicate similar amino acids.


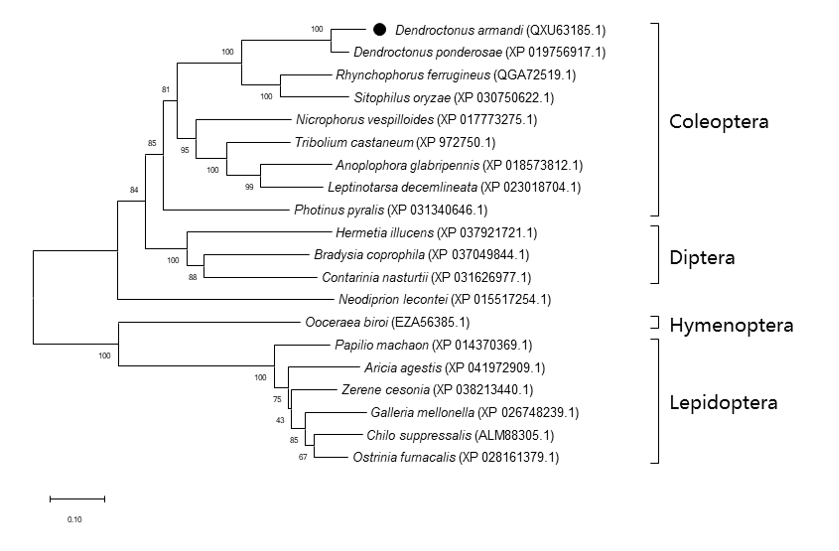


**Figure S2** Phylogenetic analysis of SKRs from other insect species. The phylogenetic tree constructed by the Maximum Likelihood method using the amino acidic substitution model WAG in MEGA 5.0. Bootstrap values after 500 pseudo-replicates are shown at nodes. The bootstrap values (in %) are given at each branch point. The black dot indicates *D. armandi* SKR.


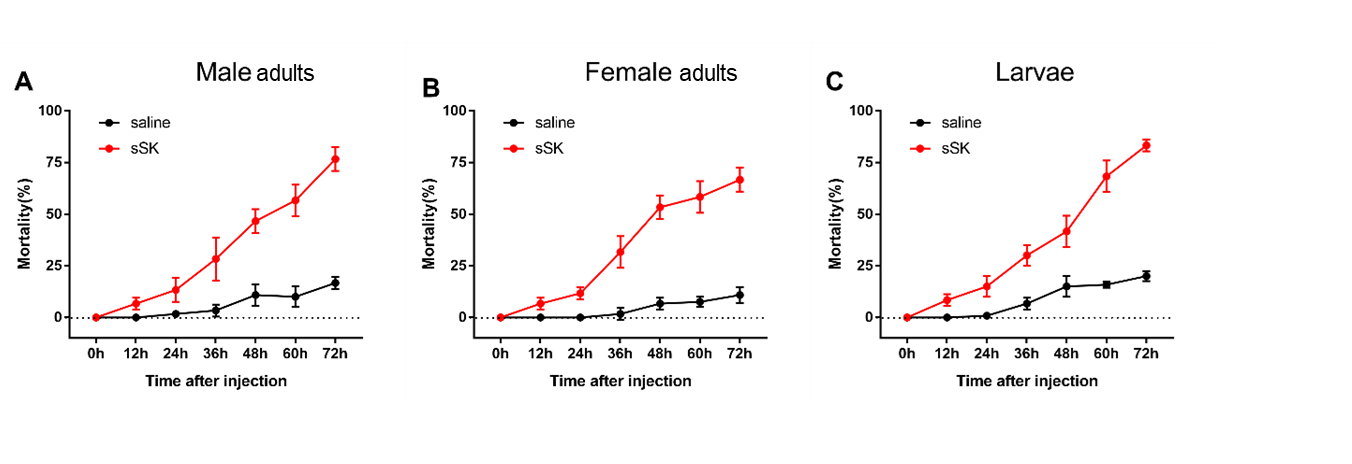


**Figure S3.** Mortality responses of RNAi *D. armandi* Male adults (A), Female adults (B) and larvae(C) at different time points after injection. Mortality responses of sSK-injected and dsGFP-injected in *D. armandi* to different time points (0, 12, 24, 36, 48, 60 and 72h). All experiments were analyzed by Student's t-test, and in (A-F) experimental beetles significantly differed (*P<* 0.01) from control.
